# The Early Prediction of Patient Outcomes in Acute Heart Failure: A Retrospective Study

**DOI:** 10.3390/jcdd12070236

**Published:** 2025-06-20

**Authors:** Maria Boesing, Justas Suchina, Giorgia Lüthi-Corridori, Fabienne Jaun, Michael Brändle, Jörg D. Leuppi

**Affiliations:** 1 University Institute of Internal Medicine, Kantonsspital Baselland, 4410 Liestal, Switzerland; 2 Faculty of Medicine, University of Basel, 4056 Basel, Switzerland; 3 Center for Executive and Continuing Education, Harvard T.H. Chan School of Public Health, Boston, MA 02115, USA; 4 Department of Internal Medicine, Kantonsspital Sankt Gallen, 9000 Sankt Gallen, Switzerland

**Keywords:** acute heart failure, risk stratification, patient outcome, mortality, intensive care, length of hospital stay, predictors, admission data

## Abstract

Background: Acute heart failure (AHF) is a major cause of hospitalizations, posing significant challenges to healthcare systems. Despite advancements in management, the rate of poor outcomes remains high globally, emphasizing the need for timely interventions. This study aimed to identify early admission-based factors predictive of poor outcomes in hospitalized AHF patients, in order to contribute to early risk stratification and optimize patient care. Methods: This retrospective single-center study analyzed routine data of adult patients hospitalized for AHF at a public university teaching hospital in Switzerland. Outcomes included in-hospital death, intensive care (ICU) treatment, and length of hospital stay (LOHS). Potential predictors were limited to routine parameters, readily available at admission. Missing predictor data was imputed and predictors were identified by means of multivariable regression analysis. Results: Data of 638 patients (median age 84 years, range 45–101 years, 50% female) were included in the study. In-hospital mortality was 7.1%, ICU admission rate 3.8%, and median LOHS was 8 days (IQR 5–12). Systolic blood pressure ≤ 100 mmHg (Odds ratio (OR) 3.8, *p* = 0.009), peripheral oxygen saturation ≤ 90% or oxygen supplementation (OR 5.9, *p* < 0.001), and peripheral edema (OR 2.7, *p* = 0.044) at hospital admission were identified as predictors of in-hospital death. Furthermore, a stroke or transient ischemic attack in the patient’s history (OR 3.2, *p* = 0.023) was associated with in-hospital death. ICU admission was associated with oxygen saturation ≤ 90% or oxygen supplementation (OR 22.9, *p* < 0.001). Factors linked to longer LOHS included oxygen saturation ≤ 90% or oxygen supplementation (IRR 1.2, *p* < 0.001), recent weight gain (IRR 1.1, *p* = 0.028), and concomitant chronic kidney disease (IRR 1.2, *p* < 0.001). Conclusions: This study validated established predictors of AHF outcomes in a Swiss cohort, highlighting the predictive value of poor perfusion status, fluid overload, and comorbidities such as chronic kidney disease. The identified predictors imply potential for developing tools to improve rapid treatment decisions. Future research should focus on the prospective external validation of the identified predictors and the design and validation of risk scores, incorporating these parameters to optimize early interventions and reduce adverse outcomes in AHF.

## 1. Introduction

Acute heart failure (AHF) is a leading cause of hospitalizations, imposing a significant burden on healthcare systems and patient quality of life [1]. The annual number of AHF hospitalizations across Europe is around 2600 per one million inhabitants, with an estimated total of 16,000 hospitalizations per year in Switzerland alone [1,2]. Despite advances in clinical management, in-hospital mortality remains high, ranging from 4% up to 26% globally [3,4,5,6,7,8]. Patients with AHF typically present to the emergency department (ED) with a variety of signs and symptoms showing congestion and hypoperfusion. The most common signs include dyspnea (including orthopnea and paroxysmal nocturnal dyspnea), recent weight gain, peripheral edema, jugular vein distention, and fatigue [9]. Patients commonly present with rales and exhibit positive hepato-jugular reflux during clinical examination [10].

Early identification of AHF patients at risk for poor outcomes presenting to an ED is crucial for optimal resource allocation, timely establishment of therapeutic interventions, and thus for improving patient prognosis. Even though considerable research has been conducted in this field, the early risk stratification of AHF patients in the acute setting remains a significant clinical challenge. Previous studies have highlighted the prognostic value of certain comorbidities, such as diabetes mellitus, chronic obstructive pulmonary disease (COPD), peripheral artery disease, and chronic kidney disease (CKD), as well as various clinical parameters, such as hemodynamic instability and impaired oxygenation [4,11,12,13,14,15,16,17]. Established risk stratification tools have proven to be effective in identifying AHF patients at high risk for poor outcomes [18,19]. Next to demographics, vital signs, clinical presentation, and patient medical history, most of these tools incorporate laboratory values such as blood urea nitrogen, serum creatinine, sodium, albumin, and natriuretic peptide [19,20,21,22].

Because the above-discussed risk stratification models depend on specific laboratory values, their application may delay the identification of high-risk patients. However, there is consensus that any unnecessary delay in treatment initiation should be avoided [10,23]. Ward et al. highlighted that delays in the administration of diuretics can prolong hospitalization, while prompt administration of furosemide, even prior to receiving certain lab results, can improve outcomes [23]. Although continuous assessment using biomarkers and other clinical parameters that are available throughout the hospitalization is indispensable [9], early recognition of high-risk patients can help to reduce delays in treatment initiation in this vulnerable patient group.

The primary objective of this study was to identify factors associated with poor outcomes in individuals with AHF, which are readily available at the time of patient admission to the ED. By focusing on parameters that can either be measured in the triage unit (vital signs, clinical examination) or gathered through a brief patient anamnesis (symptoms, major comorbidities, patient history), this study seeks to improve the understanding of early prognostic indicators and to assess if risk stratification is potentially feasible immediately upon presentation. The findings of this study may contribute to an improved early risk stratification, inform early clinical decision-making, and ultimately help to improve the management of AHF patients at high risk for poor outcomes.

## 2. Materials and Methods

### 2.1. Study Design and Setting

In this retrospective, single-center study, we analyzed clinical routine data of patients admitted to a district public hospital covering a stable population of roughly 280,000 in Switzerland. The study was previously approved by the ethics committee of northwest and central Switzerland (BASEC-ID 2022-00919).

### 2.2. Study Population

Patients aged 18 years and older who were hospitalized for at least one night at the Cantonal Hospital Baselland (Kantonsspital Baselland, KSBL) due to AHF between 2018 and 2019 and agreed to the use of their clinical routine data for research purposes were eligible for inclusion in the study. Patients for whom AHF was not the main reason for hospital admission were excluded. In case a patient was hospitalized due to AHF more than once during the study period, only the first case was included in the analysis to avoid selection bias.

In order to analyze a representative, real-world population, no further exclusion criteria were applied. In particular, patients with ventilator support, mechanical circulatory support, or under dialysis were not explicitly excluded from the study. However, extracorporeal membrane oxygenation (ECMO) is not available at KSBL; thus patients requiring immediate ECMO cannot be admitted to this hospital and hence were not included in the study.

The inclusion period (2018–2019) was chosen to ensure consistency in clinical practice and data availability. This pre-pandemic timeframe helped avoid confounding effects related to the COVID-19 pandemic, which significantly altered hospital admission patterns and treatment protocols. Furthermore, the hospital’s electronic record system underwent a major transition in 2022, limiting the comparability with more recent periods.

### 2.3. Outcomes and Predictor Variables

The outcome of primary interest was all-cause death before hospital discharge (in-hospital death). Secondary outcomes were treatment in an intensive care unit (ICU) and length of hospital stay (LOHS; defined as the number of nights from admission to alive discharge).

At the study’s institution, both hospital discharge and ICU admission decisions involve shared decision-making among healthcare providers, patients, and relatives. While individual clinician judgment and patient preferences are considered, ICU admission is typically warranted only in the presence of clearly defined criteria: the need for circulatory support due to cardiovascular instability, mechanical ventilation (invasive or non-invasive), or intensive monitoring due to conditions such as severe neurological deterioration or organ dysfunction. Hospital discharge criteria include clinical stability, no ongoing need for oxygen supplementation (unless long-term oxygen therapy has been established), and no requirement for intravenous therapies. While some degree of clinical discretion and variability across different settings remains, these structured criteria ensure that ICU admission and length of hospital stay represent largely objective and reproducible outcomes.

In line with the study objective, only parameters that are routinely available immediately after patient admission were considered as potential predictors for outcomes. These included patient sex and age, vital signs at admission, symptoms, patient clinical history and comorbidities, and the results from an orienting clinical routine examination. To reflect respiratory compromise at admission, a binary composite variable defined as either a peripheral oxygen saturation ≤ 90% or the administration of oxygen supplementation (or both) was created. Weight gain was only assumed to be present if a respective comment was made in the documentation of the patient anamnesis, without definition of a minimum threshold. Confusion was assumed to be present only if it was either explicitly documented in the admission records, or a reduced verbal response in the Glasgow Coma Scale assessment was noted. Comorbidities were assumed to be present if documented in the patient’s history, including reports from previous visits or hospitalizations; otherwise they were not assumed.

Potential predictors were carefully chosen from the available parameters based on the literature and clinical knowledge. Highly correlated or inter-dependent parameters were combined into one variable (e.g., “low oxygen saturation” and “oxygen supplementation”). To minimize risk of optimism bias and overfitting, no data-driven variable selection methods were used for the main analysis. Furthermore, the number of variables was limited to those considered most important based on the literature and clinical knowledge.

### 2.4. Data Collection and Management

Potentially eligible cases were identified from the hospital database using the relevant ICD-10 codes, as listed in Appendix A (Table A1). Cases not fulfilling the main inclusion criteria were excluded manually from this list. Basic data such as sex, age, and LOHS were directly extracted from the database. All other parameters including vital signs, symptoms, clinical examination results, medical history, and patient outcome were manually collected from the electronic patient records. Study data were collected and managed using REDCap electronic data capture tools [24].

### 2.5. Statistical Methods

Patient data were analyzed descriptively and presented as absolute and relative frequencies or median and interquartile ranges (IQRs). Covariates with up to 20% missing values were imputed using the k-nearest neighbor algorithm [25]. Variables with higher missingness were excluded from multivariable modeling; specifically, obesity was not considered due to >40% missing data. Pairwise correlations were calculated for all covariates with values ranging between −0.3 and 0.3. The dichotomous outcomes of in-hospital death and ICU treatment were modeled by means of multivariable logistic regression, using all potential predictor variables in the model. The outcome LOHS was modeled with a zero-truncated negative binomial regression on the group of patients who were discharged alive. Model stability was assessed by checking for multicollinearity via variance inflation factors, which are listed in Appendix A (Table A2).

As a first sensitivity analysis, all regression analyses were additionally performed on the original, non-imputed dataset. To address the risk of overfitting, a LASSO regression with cross-validation to correct the optimal penalty parameter was performed as a second sensitivity analysis. The results of the LASSO are added as Supplementary Materials.

**Figure A1:**
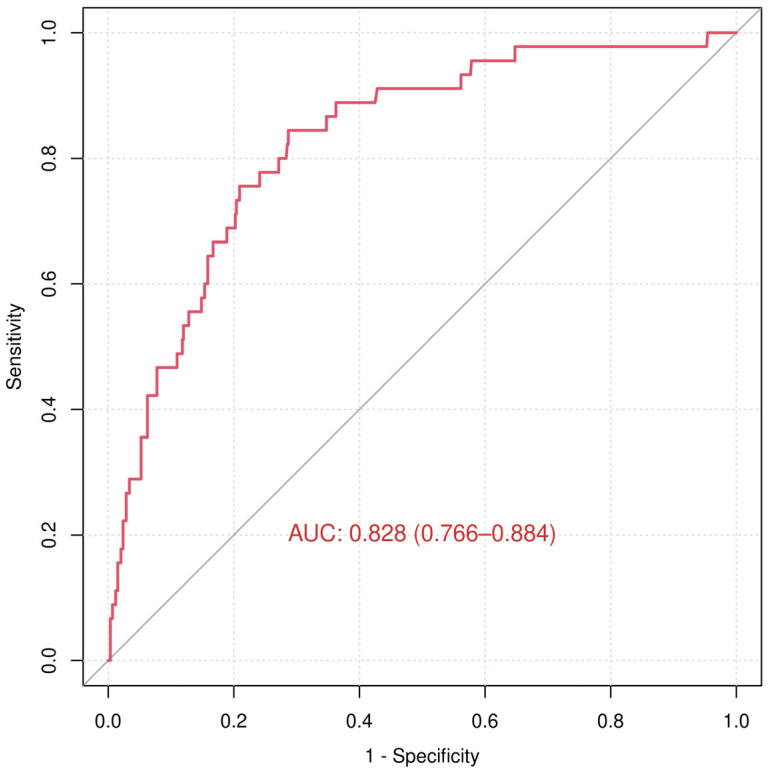
Receiver operating characteristic (ROC) curve of the multivariable model for in-hospital death with area under the curve (AUC) and 95% confidence interval.

**Figure A2:**
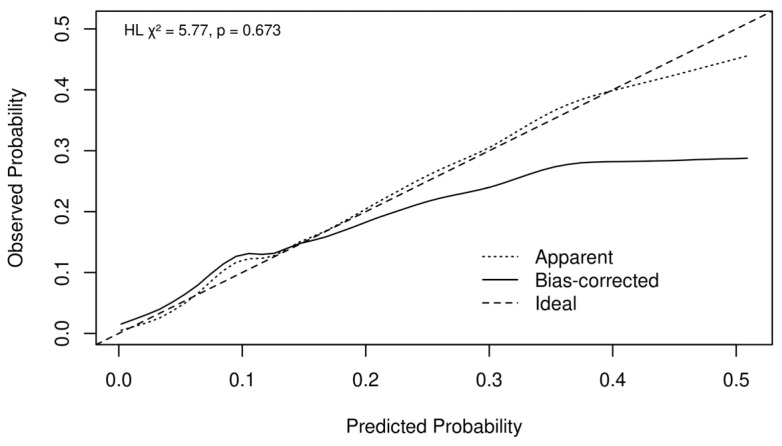
Calibration plot of the multivariable model for in-hospital death with Hosmer–Lemeshow goodness-of-fit test results (HL).

**Figure A3:**
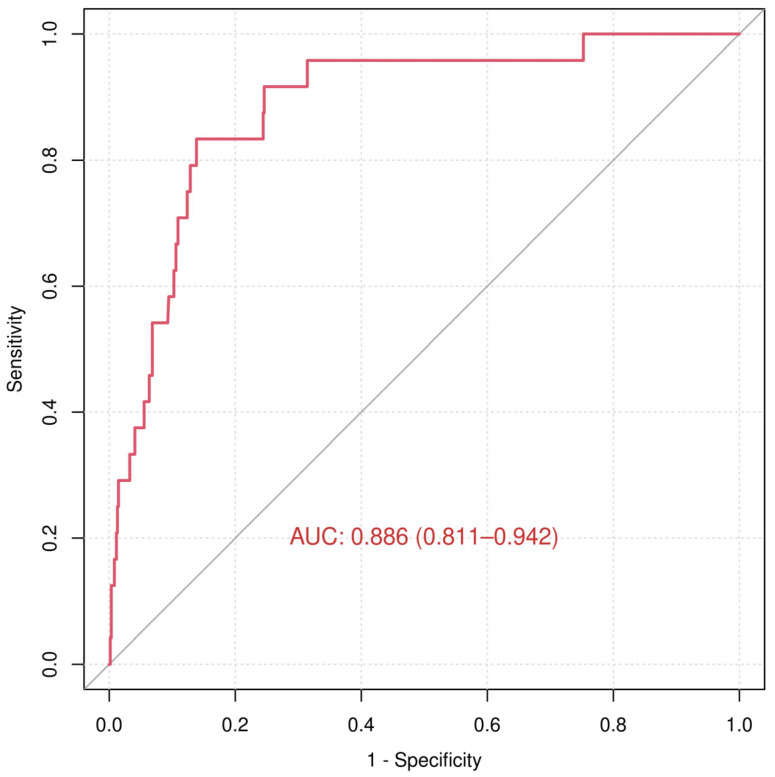
Receiver operating characteristic (ROC) curve of the multivariable model for admission to intensive care unit with area under the curve (AUC) and 95% confidence interval.

**Figure A4:**
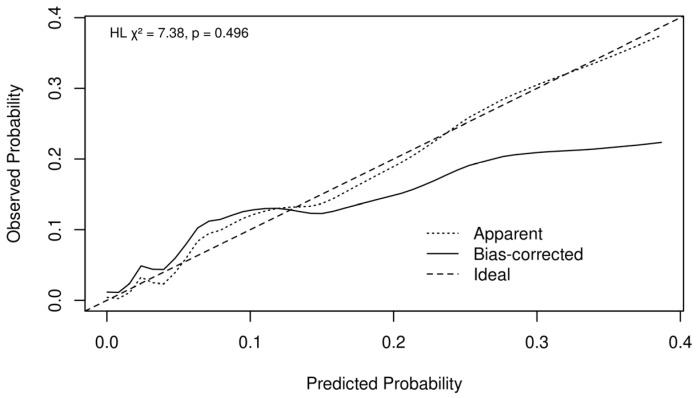
Calibration plot of the multivariable model for admission to intensive care unit with Hosmer–Lemeshow goodness-of-fit test results (HL).

To assess the internal validity of the logistic regression models, area under the receiver operating characteristic curves (AUROC) with bootstrapped 95% confidence intervals (95%-CI) were calculated. Additionally, calibration was evaluated using calibration plots and Hosmer–Lemeshow goodness-of-fit tests. Discrimination, calibration, and goodness-of-fit results are presented in Appendix A (Figure A1, Figure A2, Figure A3 and Figure A4). All reported *p*-values were two-sided at a significance level of 0.05. Data imputation and analysis were performed with R version 4.1.0 using the packages ‘bnstruct’, ‘stats’, ‘glmnet’, ‘VGAM’, ‘and ‘pROC’ [26].

## 3. Results

### 3.1. Patient Characteristics

**Figure 1:**
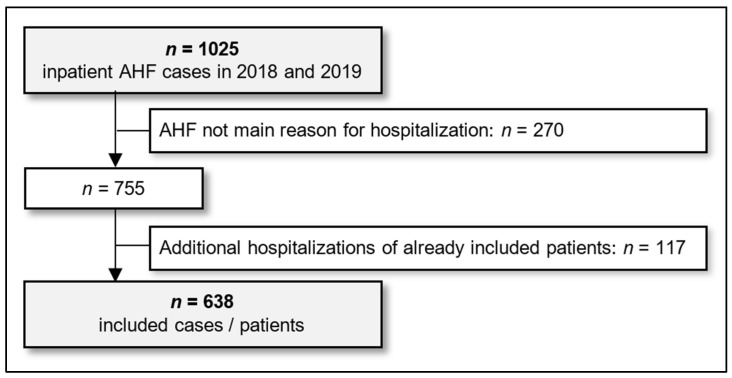
Selection of study population. AHF: acute heart failure.

In the study period, 1164 inpatient AHF cases were identified. After application of the eligibility criteria, data of 638 patients were included in the analysis. Figure 1 presents an overview of excluded cases. Characteristics of included patients are summarized in Table 1.

Patient age ranged from 45 to 101 years with men and women being equally represented in the study population. Upon admission, 42% of the patients were tachypneic and 26% already received oxygen supplementation during the admission measurement, indicating respiratory difficulties. A total of 13 patients presented with fever, indicating an accompanying infection. Most patients presented with typical signs and symptoms of AHF such as dyspnea, recent weight gain, peripheral edema, and pulmonary rales. A third heart sound was only documented in two patients, likely due to under-reporting. Similarly, documentation of the presence of a fourth heart sound and pulse irregularity was too limited for reliable reporting. Notably, the majority of patients (58%) suffered from CKD, with almost 4% of them undergoing dialysis. Patient outcomes are summarized in Table 2.

### 3.2. In-Hospital Death

**Figure 2:**
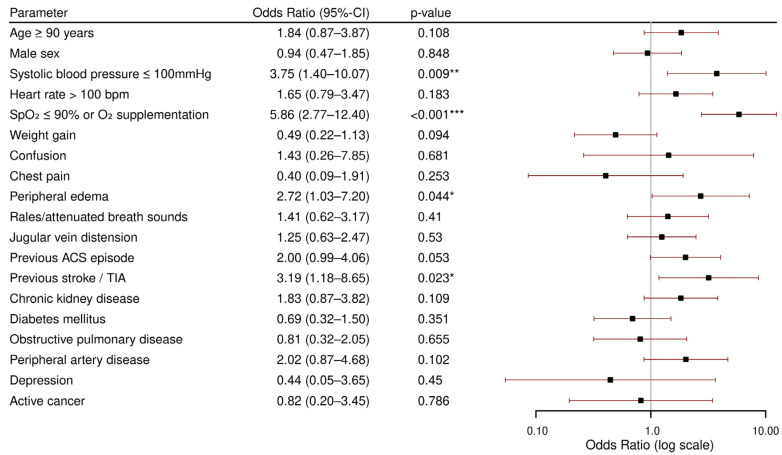
Multivariable logistic regression for in-hospital death. CI: confidence interval; bpm: beats per minute; SpO_2_: peripheral oxygen saturation; O_2_: oxygen; ACS: acute coronary syndrome; TIA: transient ischemic attack. * *p* < 0.05; ** *p* < 0.01; *** *p* < 0.001.

The results of the multivariable regression analysis modeling the odds for in-hospital death are reported in Figure 2. The odds for in-hospital death were significantly higher for patients presenting with systolic blood pressure at or below 100 mmHg (OR 3.75, *p* = 0.009), low peripheral oxygen saturation or oxygen supplementation (OR 5.86, *p* < 0.001), and peripheral edema (OR 2.72, *p* = 0.044). Moreover, a previous stroke or TIA (OR 3.19, *p* = 0.023) increased the odds of in-hospital death significantly. None of the other parameters was identified as a predictor of in-hospital death. The multivariable logistic regression model described in Figure 2 showed good internal discrimination with an AUROC of 0.83, and the Hosmer–Lemeshow test indicated no evidence of poor fit (*p* = 0.67), while the calibration plot indicated slight overestimation of in-hospital death risk (see Appendix A and Figure A1 and Figure A2).

### 3.3. Admission to Intensive Care Unit (ICU)

**Figure 3:**
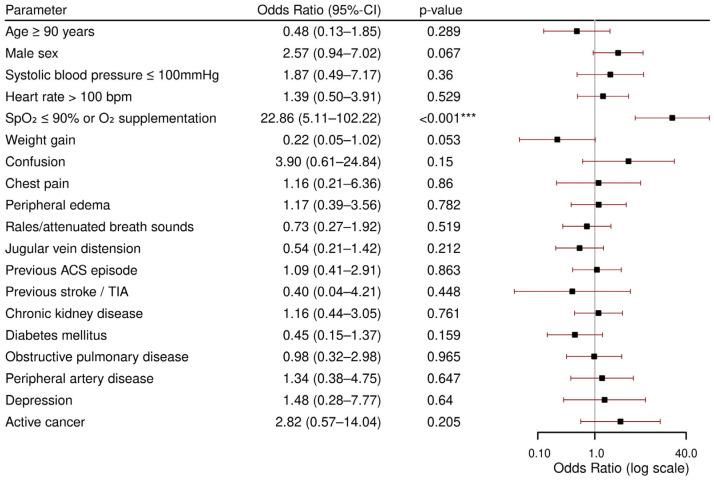
Multivariable logistic regression for intensive care unit admission. CI: confidence interval; bpm: beats per minute; SpO_2_: peripheral oxygen saturation; O_2_: oxygen; ACS: acute coronary syndrome; TIA: transient ischemic attack. *** *p* < 0.001.

Figure 3 shows the results of the multivariable regression model for ICU admission. Patients that had a low oxygen saturation or were supplemented with oxygen upon admission had the highest odds of being transferred to an ICU (OR 22.86, *p* < 0.001). None of the other parameters was found to be predictive for ICU admission. The multivariable logistic regression model showed good internal discrimination with an AUROC of 0.89, and the Hosmer–Lemeshow test indicated no evidence of poor fit (*p* = 0.50), while the calibration plot indicated slight overestimation of ICU admission risk (see Appendix A and Figure A3 and Figure A4).

### 3.4. Length of Hospital Stay (LOHS)

**Figure 4:**
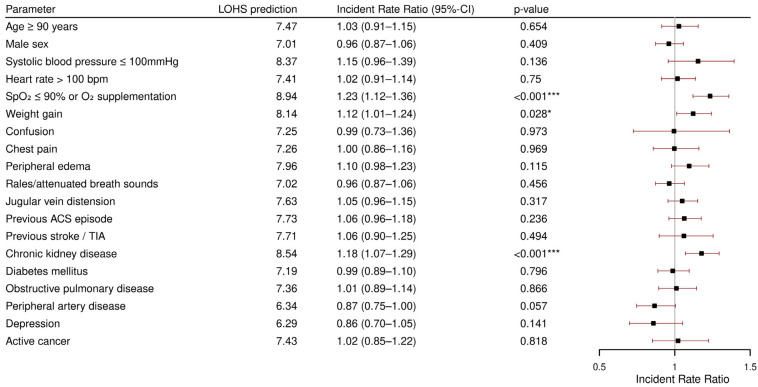
Multivariable zero-truncated negative binomial regression for length of hospital stay. LOHS: length of hospital stay; CI: confidence interval; bpm: beats per minute; SpO_2_: peripheral oxygen saturation; O_2_: oxygen; ACS: acute coronary syndrome TIA: transient ischemic attack. * *p* < 0.05; *** *p* < 0.001.

Figure 4 provides incident rate ratios for the predictors of the LOHS in the group of patients who were discharged alive (*n* = 593). The median LOHS of this patient subgroup was 8 days. The LOHS prediction at the intercept (7.4 days) is the LOHS when all included covariates are absent (for categorical covariates) or at their mean (for the continuous covariate age). The following parameters were found to have a positive association with longer LOHS: a peripheral oxygen saturation below 90% or oxygen supplementation (predicted LOHS: 8.94 days, *p* < 0.001), recent weight gain before admission (predicted LOHS: 8.14 days, *p* = 0.028), and CKD (predicted LOHS: 8.54 days, *p* < 0.001).

## 4. Discussion

In this retrospective, monocentric study, we analyzed early admission data of 638 patients hospitalized for AHF in a public hospital in Switzerland in 2018 and 2019. The main objective was to identify factors associated with poor outcomes, focusing on parameters that are available prior to, or immediately after patient admission. The study has two main findings:

1. Low systolic blood pressure, low peripheral oxygen saturation or oxygen supplementation, peripheral edema at admission, and previous stroke or TIA were independently associated with in-hospital death.
2. Patients with low peripheral oxygen saturation or oxygen supplementation at admission were more likely to experience more intensive care needs, including a higher likelihood of ICU admission and prolonged hospitalization. Additionally, patients with recent weight gain and CKD were prone to be hospitalized longer than the average patient.

### 4.1. Patient Characteristics

With a median age of 84 years, around half of the included patients being female, the majority suffering from dyspnea as well as exhibiting peripheral edema and rales at admission, and the most common non-cardiac comorbidities being CKD, anemia, and diabetes mellitus, patients’ demographics, clinical presentations, and comorbidity profiles were aligned with that of other cohorts [4,12,15,16,24,28]. In-hospital mortality in our cohort was 7.1%, and median LOHS was 8 days, both of which are comparable to findings from other European studies [4,5,15]. Overall, the study cohort appeared to be a representative sample of patients hospitalized due to AHF.

### 4.2. In-Hospital Death

The parameters identified as predictors of in-hospital death in our study are consistent with those established in the medical literature. Low systolic blood pressure, an indicator for severe cardiac dysfunction and a poor perfusion status, has long been recognized as a strong predictor of poor AHF outcome [4,11,12,17,29]. Established AHF guidelines recommend the short-term application of the calcium-sensitizer and potassium-channel-opener levosimendan in patients with poor perfusion, which can help to increase cardiac output, reduce systemic vascular resistance, and lower pulmonary capillary wedge pressure [10,30]. The early identification of high-risk patients may prompt a timely examination to further assess the perfusion status, facilitating the initiation of appropriate therapy and potentially improving prognosis. On the other hand, low peripheral oxygen saturation and the subsequent need for oxygen supplementation reflect the severity of respiratory compromise, often resulting from pulmonary congestion and peripheral hypoperfusion [10]. Both have previously been shown to predict short-term AHF mortality [17,31,32]. Established tools, such as the ‘Emergency Heart Failure Mortality Risk Grade’ (EHMRG) and the ‘Multiple Estimation of Risk Based on the Emergency Department Spanish Score in Patients with AHF’ (MEESSI-AHF) rely on systolic blood pressure and oxygenation status for early risk stratification in AHF patients [18,22]. These tools were specifically designed to support frontline clinicians in identifying high-risk AHF patients early and aid in decisions regarding level of care, hospital admission, and monitoring intensity. Peripheral edema, a common sign of volume overload and right heart failure, was the only symptom that was associated with in-hospital mortality in our cohort, with affected patients having a 2.7-fold risk of dying during their hospitalization [9,33]. Shoaib et al., who investigated the outcomes of over 120,000 AHF patients in England and Wales, reported that patients with peripheral edema had a significantly higher risk of in-hospital death than those without, and the risk increased even further with the edema severity [34]. With peripheral edema being such a common symptom—over three quarters were affected in our cohort—these results suggest that an objective edema severity grading might further improve early risk stratification. Regarding patient history and comorbidities, patients with a previous stroke or TIA had 3.2-fold in-hospital mortality odds compared to those without. Even though patients with a previous stroke or TIA were relatively rare in the study cohort, these numbers are compelling and align with previous studies [12,13,14]. Unlike the previously discussed vital signs and symptoms, comorbidities and events in the patient’s history are not directly linked to the current AHF severity and consequently not in the focus of early AHF management. However, the substantially increased mortality risk of affected patients suggests that this condition should be given special attention.

Notably, the multivariable model did not identify age ≥ 90 years as a predictor of in-hospital death. This result seems surprising, since older age has proven to have strong predictive value for in-hospital mortality in a wide range of conditions and settings [17,35,36]. Furthermore, age is one of the factors implemented in established AHF risk-stratification tools, such as the EHMRG and the MEESSI-AHF [18,22]. In our study cohort, the unadjusted in-hospital mortality was only 4.2% in the youngest quartile (<77 years) and 11.3% in the oldest quartile (>89 years) of patients, suggesting an age-related gradient. This mortality differential is clinically relevant and highlights the importance of age as a risk factor in AHF. However, the non-significance of age ≥ 90 as a predictor in our multivariable model may reflect the presence of more proximate clinical indicators that frequently co-occur with advanced age and may better capture acute deterioration. It is also plausible that individualized treatment decisions based on mid- to long-term prognosis may have resulted in less-intensive treatment strategies and, consequently, higher mortality rates in the oldest patients. This supports the idea that while age alone is an important contextual factor, it may not fully capture short-term mortality risk when more acute clinical parameters are available. Nevertheless, given the significantly higher mortality observed in this group, very old patients may still benefit from tailored risk stratification and targeted management strategies. The use of more granular age-categories, such as those implemented in the MEESSI-AHF or by De Matteis et al., may help to map this relationship and should thus be considered in future tools for early AHF risk stratification [3,22].

Similarly, diabetes mellitus, a well-established risk factor in AHF short- and long-term outcomes, did not emerge as a significant predictor of in-hospital mortality [22,37,38,39]. This finding may be influenced by specific characteristics of the study population and the relatively small number of events, which could limit the power to detect associations with some comorbidities. In fact, mortality within the group of patients with concomitant diabetes mellitus was even slightly lower than in the overall population (6.6% vs. 7.1%). Consequently, this highlights a potential limitation in the generalizability of the presented results, suggesting that the impact of diabetes mellitus on mortality may vary across different cohorts and warrants further investigation in larger, more diverse populations.

Our findings support the potential applicability of established tools like the MEESSI-AHF and the EHMRG beyond risk stratification to informing early treatment decisions. Implementation studies of the MEESSI-AHF have shown that, while the tool aligns well with physician judgment, its use alone does not necessarily improve patient outcomes, as disposition decisions are multifactorial and often overruled for clinical reasons beyond HF severity [39,40,41]. The EHMRG on the other hand has proven to outperform physicians’ judgment in identifying high-risk patients and thus help potentially avoiding inappropriate discharge and ultimately reduce mortality [18,42,43,44]. However, there is consensus that these tools can be used to supplement, but should be integrated with comprehensive clinical assessment and not used in isolation [9,19].

### 4.3. ICU Admission

Patients admitted with a low peripheral oxygen saturation (≤90%) or oxygen supplementation had almost 23-fold odds of being admitted to an ICU. The strong association is likely explained by the fact that oxygen supplementation is typically administered in patients with an initial peripheral oxygen saturation below 90%, which in itself is an indication, as opposed to a predictor, of ICU treatment in AHF [45]. This introduces a potential risk of circularity in interpretation, as oxygen supplementation may reflect both disease severity and influence clinical decisions regarding ICU admission. Indeed, out of the 24 patients that were admitted to ICU, only 3 had not received oxygen supplementation at admission, suggesting that ICU treatment was predominantly driven by respiratory compromise at presentation. These findings imply that oxygen supplementation functions more as a surrogate marker of severity, rather than an independent risk factor. Nevertheless, the inclusion of the composite parameter combining low peripheral oxygen saturation (≤90%) with oxygen supplementation remains clinically meaningful for early risk stratification, as the combination identifies patients requiring immediate respiratory support at presentation. An additional sensitivity analysis using the non-composite parameter ‘peripheral oxygen saturation ≤ 90%’ instead of the composite parameter resulted in slightly lower model discrimination (AUROC 0.79), yet the parameter remained the sole significant predictor for ICU admission in the multivariable model, underscoring the robustness of hypoxemia as a driver of critical illness in this context. The numbers also suggest that the most critical state of the respiratory situation is commonly present at the point of admission, while clinical management can effectively improve the respiratory status.

It needs to be noted that variations in ICU admission policies and treatment protocols across different institutions, particularly in low-to-middle-income settings with limited resources, may further affect the generalizability of our findings, underscoring the need for context-specific validation of risk stratification tools.

### 4.4. Length of Hospital Stay (LOHS)

The result that low peripheral oxygen saturation or oxygen supplementation at admission was associated with longer LOHS goes in line with the previously discussed results in Section 4.2 and Section 4.3. Both low peripheral oxygen saturation and oxygen supplementation reflect a poor respiratory status. The results underline the importance of respiratory parameters to be implemented in early risk stratification tools, regardless of the specific outcome of interest. Weight gain has previously been shown to be predictive of adverse AHF outcomes, in particular longer hospital stays [46]. Our findings confirm these results, which suggest that patients with significant fluid overload and congestion require longer diuretic therapy and longer monitoring periods to achieve euvolemia, thereby being subject to longer hospital stays. Similarly, CKD is a well-documented risk factor for prolonged hospital stays in AHF [46,47,48]. The presence of impaired renal function is a source of increased complexity in HF management, due to a complex bidirectional relationship with altered pharmacokinetics, increased risk of toxicities of HF therapeutics, and impaired response to diuretics [49,50]. Furthermore, CKD is often an exclusion criterion in clinical trials for AHF management, which leads to the lack of robust evidence of heart failure management in the affected patient group [38,49]. Considering the fact that CKD was not found to be predictive of in-hospital death and intensive care in our cohort, these findings may indicate that CKD does not directly increase the severity of AHF. Instead, it may result in less aggressive and less effective management due to the above-described factors, potentially leading to longer hospital stays.

### 4.5. Implications for Clinical Practice

Generally, the early identification of AHF patients at high risk of poor outcomes could help prioritize additional assessments and timely therapeutic interventions. The predictors that were confirmed in this study are all readily available at the time of hospital admission and can therefore support early risk stratification in patients with AHF. In real-world clinical settings, these variables may inform early therapeutic decision-making by identifying patients who are likely to deteriorate and thus require close monitoring, early respiratory support, or escalation of care. For instance, identifying patients at high risk for in-hospital mortality could prompt immediate evaluation for hemodynamic compromise and guide interventions, such as early vasodilator or inotrope administration [9]. Similarly, in patients presenting with signs of congestion or respiratory compromise, prompt initiation of intravenous diuretics may improve symptom relief and reduce the risk of clinical deterioration [10,50]. These practical insights can help prioritize resource allocation, inform triage decisions, and shape multidisciplinary care pathways, particularly in high-volume or resource-limited settings.

Ultimately, incorporating these readily assessable risk factors into clinical scores or decision-support tools could improve early identification of high-risk patients and facilitate more targeted and timely interventions.

### 4.6. Limitations and Strengths

Our study has some limitations. First, the single-center design with a relatively homogenous population of 638 Swiss patients may have introduced selection bias limiting the generalizability of the results to broader and more diverse settings. Our study did not include a comparison group of patients without AHF or with different management strategies, which limits external comparability. Moreover, the study population did not include patients with AHF requiring ECMO or other forms of mechanical circulatory support, which reflects a deliberate focus on the broader AHF population, typically encountered in routine care and limits the external validity of the findings for the most critically ill subset of AHF patients. However, the results remain clinically relevant and applicable to a large proportion of patients with AHF in routine clinical settings and support early risk stratification in patients who do not require mechanical circulatory support. Second, being a retrospective study with clinical routine data, no conclusions regarding causality can be drawn. Furthermore, due to the retrospective design, data quality relied strongly on accurate documentation in patient records, which may have resulted in incomplete information. In particular, symptoms and comorbidities that were not documented in patients’ records were assumed not to be present but may have been under-reported (e.g., presence of third heart sound). Additionally, missing values in the vital signs, despite being imputed, might have affected the robustness of the results. However, sensitivity analyses of the non-imputed dataset yielded consistent findings. With the low number of events (45 in-hospital deaths, 24 ICU admissions), a standard logistic regression analysis carries the risk of multicollinearity and overfitting. But additional analyses including the assessment of variance inflation factors and penalized regression analysis confirmed the robustness of the findings.

More detailed admission parameters such as the New York Heart Association classification for dyspnea, irregular pulse, presence of third or fourth heart sound, and additional risk factors such as previous AHF hospitalizations, obesity, smoking status, and alcohol consumption could potentially add predictive value to the models, but could not be included in the analyses due to incomplete documentation. Similarly, stratification by left ventricular ejection fraction or heart failure subtype was not possible due to insufficient and incomplete data, which may limit the generalizability of our findings across different heart failure phenotypes. In particular, due to a missingness rate exceeding 40%, the well-known risk factor obesity was excluded from the multivariable models to avoid the risk of biased imputation. However, its absence may have led to biased model estimates, as other variables that often coexist with obesity, such as diabetes, hypertension, or CKD may have inadvertently captured some of its effect. This phenomenon could mask the independent role of obesity and its omission may affect model accuracy and applicability, especially in populations with high obesity prevalence.

While the early identification of high-risk patients can promote an early treatment initiation, some patient groups—especially those with accompanying conditions such as ACS—require additional diagnostic steps in order to initiate the appropriate treatment. The accurate assessment of AHF severity requires a comprehensive evaluation, including laboratory and echocardiography findings, as well as consideration of the underlying etiology and HF subtype. These limitations have to be kept in mind when applying the results in clinical practice.

Despite the described limitations, our study also has several strengths. First, we maintained a clear and strict focus on predictors available before or immediately upon admission, which enhances the clinical applicability of our findings for early risk stratification. Second, the study is based on high data accuracy and granularity, particularly for variables often unavailable in structured databases, such as clinical symptoms, comorbidities, anamnesis, and admission findings, enabling robust insights from a carefully curated cohort of 638 patients with minimal information bias. Third, we conducted a thorough statistical analysis, including sensitivity analyses, which confirmed the consistency and robustness of our findings.

Altogether, our study provides valuable insights into the predictors of outcomes in hospitalized AHF patients, which can inform clinical decision-making and improve early risk stratification.

## 5. Conclusions

While the identified predictors of patient outcomes in AHF align with the existing literature, our study validated these established predictors in a Swiss cohort, emphasizing their local relevance. The findings underscore the predictive value of a poor perfusion status, symptoms of fluid overload, and comorbidities such as CKD for AHF outcome. The variation in the identified predictors, depending on the investigated outcome, reflects the complexity of early risk stratification in AHF. The prediction of adverse outcomes remains challenging due to their dependence on individual patient preferences (particularly for ICU treatment) and clinical management decisions made during hospitalization. Nonetheless, by focusing on data obtainable in the triage or early assessment phase (including vital signs, clinical examination, symptoms, and patient history), the results of this study highlight the practical feasibility of risk stratification at the point of care, before laboratory or imaging results are available. The early availability of independent predictors suggests the potential for integrating these early indicators into pragmatic clinical decision-support tools, which could assist healthcare professionals in quickly identifying high-risk AHF patients, optimizing resource allocation, and ultimately improving outcomes through timely interventions. Such tools should incorporate key parameters, like oxygenation status, perfusion status, peripheral edema, and concomitant CKD and could trigger early, intense monitoring and the timely initiation of additional diagnostic steps in order to guide appropriate treatment at predefined thresholds. Furthermore, early respiratory support for patients who do not meet ICU criteria yet but are identified as “high-risk” may prevent further respiratory deterioration. In addition, the early identification of patients at high risk for long hospital stays could benefit from early, multidisciplinary discharge planning to prevent unnecessary delays.

The early risk stratification of AHF patients is crucial, as rapid treatment initiation is important for high-risk patients. Effective risk stratification tools, such as scores, can streamline clinical decision-making processes and ensure that high-risk patients receive immediate intervention, potentially reducing adverse outcomes in AHF. Importantly, the results of this study should not be generalized to the most critically ill AHF patients, such as those requiring ECMO or mechanical circulatory support, as these were not represented in our cohort. Furthermore, the rather homogenous study population from one specific healthcare setting limits generalizability. However, the reported findings remain relevant to a large population of AHF patients typically encountered in routine clinical practice in Europe. Future research and multicentric studies should focus on the prospective and external validation of the reported findings in different healthcare settings and the integration of the identified predictors into triaging pathways. Furthermore, the development and validation of robust risk stratification tools, in conjunction with more detailed clinical evaluations, are essential to improve early identification and management of high-risk AHF patients. Prospective studies could enable stratification by heart failure subtype and the inclusion of additional factors such as NYHA functional class, obesity, and smoking status.

## Figures and Tables

**Table 1:** Patient characteristics.

		Overall (*n* = 638)	Missing (%)
Demographics	Age (years), median [IQR] (range)	84 [77, 89] (45–101)	-
	Male sex, *n* (%)	320 (50.2)	-
Vital signs at admission	Systolic BP (mmHg), median [IQR]	135 [118, 153]	2.0
	Diastolic BP (mmHg), median [IQR]	79 [69, 90]	2.0
	Heart rate (bpm), median [IQR]	84 [70, 100]	1.6
	Peripheral O_2_ saturation %, median [IQR]	94 [91, 96]	4.2
	Respiratory rate ≥ 22 brpm, *n* (%)	215 (41.8)	19.4
	O_2_ supplementation, *n* (%)	168 (26.3)	-
	Fever (body temperature ≥ 38 °C)	13 (2.2)	7.2
Symptoms at admission,	Dyspnea	556 (87.1)	-
*n* (%)	Weight gain	194 (30.4)	-
	Fatigue	128 (20.1)	-
	Chest pain	65 (10.2)	-
	Confusion	15 (2.4)	-
	Nycturia	21 (3.3)	-
	Nocturnal cough	16 (2.5)	-
Clinical examination,	Peripheral edema	486 (76.2)	-
*n* (%)	Pulmonary rales	351 (55.0)	-
	Jugular vein distension	301 (47.2)	-
	Hepato-jugular reflux	41 (6.4)	-
	Attenuated breath sounds	126 (19.7)	-
Cardiac medical history,	Previously diagnosed heart failure	505 (79.2)	-
*n* (%)	GDMT for heart failure ^1^ (*n* = 505)	378 (74.9)	-
	Previous hospitalization for AHF ^2^ (*n* = 505)	128 (25.3)	-
	Previous ACS episode	217 (34.0)	-
	Pacemaker or ICD	87 (13.6)	-
	Previous CRT	6 (0.9)	-
	Previous valvular surgery	38 (6.0)	-
	Mechanical circulatory support (IABP, VAD)	0	-
Comorbidities,	Arterial hypertension	487 (76.3)	-
*n* (%)	Valvular heart disease	288 (45.1)	-
	Coronary artery disease	238 (37.3)	-
	Atrial fibrillation	374 (58.6)	-
	Chronic kidney disease	370 (58.0)	-
	Anemia	207 (32.4)	-
	Diabetes mellitus	196 (30.7)	-
	Obstructive pulmonary disease	107 (16.8)	-
	Peripheral artery disease	77 (12.1)	-
	Previous stroke or TIA	54 (8.5)	-
	Depression	35 (5.5)	
	Active cancer	44 (6.9)	-
	Obesity	106 (29.9)	44.4

^1^ According to guidelines from the study period [27]. ^2^ Only hospitalizations in the same institution were assessed. IQR: interquartile range; BP: blood pressure; bpm: beats per minute; brpm: breaths per minute; GDMT: guideline-directed medical therapy; AHF: acute heart failure; ACS: acute coronary syndrome; ICD: implantable cardioverter defibrillator; CRT: cardiac resynchronization therapy; IABP: intra-aortic balloon pump; VAD: ventricular assist device; TIA: transient ischemic attack.

**Table 2:** Patient Outcomes.

Outcome	
In-hospital death, *n* (%)	45 (7.1)
Admission to intensive care unit, *n* (%)	24 (3.8)
Length of hospital stay (nights), median [IQR] ^1^	8 [5, 12]

^1^ Patients who were discharged alive, *n* = 593. IQR: Interquartile range.

## Data Availability

The data presented in this study are available from the corresponding author upon reasonable request. The data are not publicly available due to restrictions in data privacy.

## Supplementary Materials

The following supporting information can be downloaded at https://www.mdpi.com/article/10.3390/jcdd12070236/s1: Table S1: LASSO regression results with optimal lambda for in-hospital death. Table S2: LASSO regression results with optimal lambda for intensive care unit admission. Table S3: LASSO regression results with optimal lambda length of hospital stay.

## Abbreviations

The following abbreviations are used in this manuscript:

ACS	Acute coronary syndrome
AHF	Acute heart failure
AUROC	Area under the receiver operating characteristic curves
BP	Blood pressure
bpm	Beats per minute
brpm	Breaths per minute
CI	Confidence interval
CKD	Chronic kidney disease
COPD	Chronic obstructive pulmonary disease
CRT	Cardiac resynchronization therapy
ED	Emergency department
EHMRG	Emergency Heart Failure Mortality Risk Grade
ICD	Implantable cardioverter defibrillator
ICU	Intensive care unit
IQR	Interquartile range
LOHS	Length of hospital stay
MEESSI-AHF	Multiple Estimation of Risk Based on the Emergency Department Spanish Score in Patients with AHF
OR	Odds ratio
TIA	Transient ischemic attack

## Appendix A

**Table A1:** ICD-10 codes for acute heart failure.

ICD-10 Code	Diagnosis Description
I11.0	Hypertensive heart disease with (congestive) heart failure
I13.0	Hypertensive heart and kidney disease with (congestive) heart failure.
I50.0	Congestive heart failure
I50.1	Left ventricular failure
I50.2	Systolic heart failure
I50.3	Diastolic heart failure
I50.4	Combined systolic and diastolic heart failure
I50.8	Other heart failure
I50.9	Unspecified heart failure

**Table A2:** Variance inflation factors of multivariable logistic regression models for in-hospital death and ICU admission.

Parameter	VIF In-Hospital Death	VIF ICU Admission
Age ≥ 90 years	1.14	1.07
Male sex	1.10	1.14
Systolic blood pressure ≤ 100 mmHg	1.16	1.18
Heart rate > 100 bpm	1.07	1.10
SpO_2_ ≤ 90% or O_2_ supplementation	1.07	1.04
Weight gain	1.14	1.05
Confusion	1.04	1.12
Chest pain	1.07	1.19
Peripheral edema	1.13	1.20
Rales/attenuated breath sounds	1.10	1.11
Jugular vein distension	1.10	1.09
Previous ACS episode	1.16	1.19
Previous stroke/TIA	1.11	1.15
Chronic kidney disease	1.09	1.17
Diabetes mellitus	1.18	1.13
Obstructive pulmonary disease	1.11	1.17
Peripheral artery disease	1.10	1.10
Depression	1.03	1.08
Active cancer	1.15	1.30

VIF: variance inflation factor; bpm: beats per minute; SpO_2_: peripheral oxygen saturation; O_2_: oxygen; ACS: acute coronary syndrome; TIA: transient ischemic attack.
