# Supplementary material for: The Early Prediction of Patient Outcomes in Acute Heart Failure: A Retrospective Study
Source: J Cardiovasc Dev Dis. 2025 Jun 20;12(7):236. doi: 10.3390/jcdd12070236 (PMC12295718; doi:10.3390/jcdd12070236)
Supplement: Supplementary file 1 [file jcdd-12-00236-s001.zip › Supplementary_Material_JCDD.pdf]

# Supplementary Material

## The Early Prediction of Patient Outcome in Acute Heart Failure: A retrospective study.

**Table S1:** LASSO regression results with optimal lambda for in-hospital death.

| LASSO selected parameter | Odds Ratio (95%-CI) | p-value |
|---|---|---|
| Age (years) ≥ 90 | 2.16 (1.06 - 4.42) | 0.034* |
| Systolic blood pressure ≤ 100mmHg | 3.57 (1.40 - 9.08) | 0.008** |
| Heart rate > 100 bpm | 1.76 (0.85 - 3.61) | 0.126 |
| SpO2 ≤ 90% or O2 supplementation | 5.87 (2.82 - 12.25) | <0.001*** |
| Weight gain | 0.54 (0.24 - 1.21) | 0.135 |
| Chest pain | 0.41 (0.09 - 1.87) | 0.25 |
| Peripheral edema | 2.62 (1.02 - 6.75) | 0.045* |
| Previous ACS episode | 1.87 (0.95 - 3.68) | 0.07 |
| Previous stroke / TIA | 3.16 (1.21 - 8.28) | 0.019* |
| Chronic kidney disease | 1.81 (0.88 - 3.71) | 0.107 |
| Peripheral artery disease | 1.86 (0.82 - 4.23) | 0.138 |

**Table S2:** LASSO regression results with optimal lambda for intensive care unit admission.

| LASSO selected parameter | Odds Ratio (95%-CI) | p-value |
|---|---|---|
| Male sex | 3.11 (1.21 - 7.98) | 0.019* |
| Systolic blood pressure ≤ 100mmHg | 2.54 (0.74 - 8.68) | 0.136 |
| SpO2 ≤ 90% or O2 supplementation | 20.98 (4.82 - 91.44) | <0.001*** |
| Weight gain | 0.23 (0.05 - 1.04) | 0.056 |
| Confusion | 3.76 (0.65 - 21.63) | 0.138 |
| Jugular vein distension | 0.56 (0.23 - 1.40) | 0.218 |

**Table S3:** LASSO regression results with optimal lambda length of hospital stay.

| LASSO selected parameter | Incident rate ratio (95%-CI) | p-value |
|---|---|---|
| Systolic blood pressure ≤ 100mmHg | 1.09 (0.76 - 1.56) | 0.363 |
| SpO2 ≤ 90% or O2 supplementation | 1.19 (0.98 - 1.43) | <0.001*** |
| Weight gain | 1.15 (0.93 - 1.41) | 0.01** |
| Peripheral edema | 1.06 (0.85 - 1.33) | 0.293 |
| Rales or attenuated breath | 0.94 (0.77 - 1.15) | 0.233 |
| Jugular vein distension | 1.07 (0.89 - 1.29) | 0.148 |
| Previous ACS episode | 1.08 (0.89 - 1.31) | 0.127 |
| Chronic kidney disease | 1.16 (0.96 - 1.40) | 0.003** |
| Peripheral artery disease | 0.85 (0.64 - 1.13) | 0.026* |
| Depression | 0.88 (0.58 - 1.33) | 0.229 |
